# Estimating prevalence of child and youth mental disorder and mental health-related service contacts: a comparison of survey data and linked administrative health data

**DOI:** 10.1017/S204579602200018X

**Published:** 2022-05-19

**Authors:** L. Duncan, K. Georgiades, L. Wang, J. Edwards, J. Comeau

**Affiliations:** Offord Centre for Child Studies, Psychiatry & Behavioural Neurosciences, McMaster University, Hamilton, ON, Canada

**Keywords:** Administrative data, epidemiology, health services, population surveys, prevalence

## Abstract

**Aims:**

Prevalence estimates of child and youth mental disorder and mental health-related service contacts are needed for policy formulation, research, advocacy and resource allocation. Our aim is to compare prevalence estimates of child and youth mental disorder and mental health-related service contacts derived from general population survey data *v*. linked administrative health data.

**Methods:**

Provincially representative 2014 Ontario Child Health Study data were linked to administrative health records for 5563 children and youth aged 4–17 in Ontario. Emotional disorders (mood and anxiety) and attention-deficit/hyperactivity disorder were assessed using a standardised diagnostic interview in the survey and using diagnostic codes in administrative health data. Physician-based mental health-related service contacts were assessed using parent self-reports from the survey and administrative data related to mental health-related diagnostic codes. Prevalence estimates were calculated and compared based on one-sample *z*-tests and ratios of survey data to administrative data-based prevalence. Sensitivity, specificity and agreement between classifications were compared using *κ*. Prevalence estimates were calculated by age, sex and geography sub-groups and consistent group differences across data source were counted.

**Results:**

Disorder prevalence and service contact estimates were significantly higher in survey data in all cases, except for mood disorder. Ratios of survey data to administrative data-based prevalence varied, ranging from 0.80 (mood) to 11.01 (attention-deficit/hyperactivity disorder). Specificity was high (0.98–1.00), sensitivity was low (0.07–0.41) and agreement ranged from slight (*κ* = 0.13) to moderate (*κ* = 0.46). Out of 18 sub-group difference comparisons, half were non-significant in either data source. In the remaining nine comparisons, the only significant differences between groups that were consistent across data source were for sex-based differences (attention-deficit/hyperactivity disorder and service contacts). There were no consistent age- or geography-based differences in prevalence across data sources.

**Conclusions:**

Our findings suggest that conclusions drawn about prevalence, service contacts and sub-group differences in these estimates are dependent on data source. Further research is needed to understand who and what is being captured by each source. Researchers should conduct data linkage where possible to access and compare multiple sources of information.

## Introduction

Mental disorders affect one in five children and youth worldwide (Polanczyk *et al*., 2015) and lead to individual and social burdens (Waddell *et al*., 2018), and adverse outcomes (Erskine *et al*., 2016; Ploubidis *et al*., 2021). Documenting the prevalence of mental disorder among children (age 4–11) and youth (age 12–17) and the extent to which children/youth are in contact with mental health services is critical if we are to monitor the size of the problem and design effective policies to address need.

To inform equitable policy, accurate and timely estimates are needed of the number of children/youth: (1) living with a mental disorder (known prevalence); (2) in contact with mental health services (known service contacts); and (3) living with a disorder who are not in contact with mental health services (unmet need). Examining differences across population sub-groups and geographic areas reveals important patterns of disorder and service contacts (Kessler *et al*., 2012, ; Costello *et al*., 2014; Cairney *et al*., 2015; Georgiades *et al*., 2019). This information provides critical evidence for examining patterns and correlates of mental health need, temporal trends, economic analysis of service costs and the individual/social/economic impact of mental disorders.

General population epidemiological surveys and administrative health records are used to estimate population-level disorder prevalence and mental health-related service contacts. Population health surveys (herein survey data) include standardised measurement of common mental disorders and random sampling approaches to produce representative estimates. They contain detailed demographic information and include both service users and non-users enabling more precise estimation of population prevalence (Merikangas *et al*., 2010; Pitchforth *et al*., 2019). Large-scale surveys are also expensive to implement, experiencing dwindling response rates (Luiten *et al*., 2020), and are not conducted with the same frequency or geographic coverage as administrative data (Boyle *et al*., 2019).

Administrative health data (herein administrative data) are used for population monitoring/surveillance, despite not being collected for this purpose (Birkhead *et al*., 2015). Collected routinely in Canadian publicly funded health systems and as part of mandated record-keeping processes, administrative data include all individuals who have been in contact with physician-based health services and comprehensive coverage of number and type of diagnoses – including rare conditions often excluded from survey data. Limitations include (a) no standardised and validated approach to classifying individuals with disorder (Hinds *et al*., 2016); (b) availability of age, sex and postal code only as demographic characteristics; and (c) disorder classification conditional on service contact (i.e. an individual requires an administrative database record for a diagnostic code to be present) (Gandhi *et al*., 2016).

Attempts to validate the use of administrative data to estimate adult mental disorder across jurisdictions have concluded that it is suitable for surveillance, but has limitations (Kisely *et al*., 2009*a*, 2009*b*; Doktorchik *et al*., 2019). Studies comparing mood and anxiety disorder prevalence in administrative *v*. survey data found differences in prevalence and low concordance (O'Donnell *et al*., 2016; Edwards *et al*., 2020). Purported reasons for differences were that disorders were captured differently depending on the stage of illness and treatment. Other work has found large discrepancies between mental health service contacts from administrative data and both self-reported contacts (Drapeau *et al*., 2011; Palin *et al*., 2011) and chart abstractions (Steele *et al*., 2004) – pointing to social desirability and recall bias as possible reasons for low levels of agreement. No studies have considered whether between-group differences are consistent, even if overall prevalence estimates differ between administrative and survey data.

This study is the first to compare child/youth mental disorder prevalence and physician-based mental health-related service contact estimates from survey and administrative data. By describing and documenting the level of agreement between these data sources, this work is a first step and a pre-requisite to future work aimed at making recommendations about the appropriate use of different data sources for specific purposes. Study objectives are to evaluate differences in overall prevalence and sub-group estimates of individual and grouped classifications of disorder, and mental health-related service contacts. We address the following questions: (1) Are disorder prevalence and service contact estimates different by data source? (2) What is the level of agreement between classifications of disorder and service contacts? (3) Are patterns of population sub-group estimates based on age, sex and geography consistent? The information generated will help determine how our conclusions may differ depending on data source and the potential usefulness of the respective data in estimating prevalence of child/youth mental disorder and mental health-related service contacts.

## Method

### Data

**Fig. 1.**
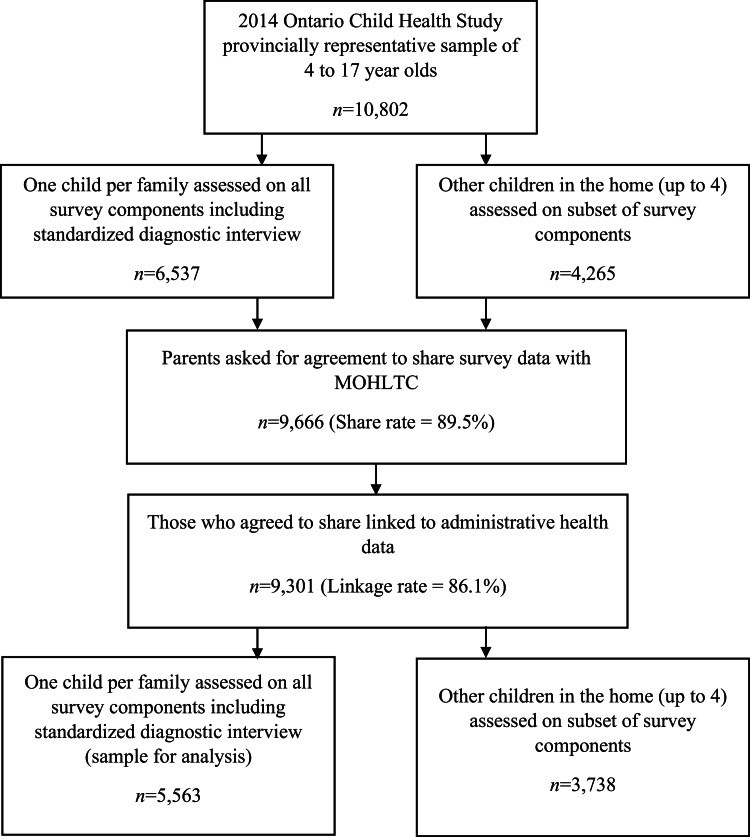
Diagram of sample selection.

This study uses provincially representative, cross-sectional 2014 Ontario Child Health Study (OCHS) data (Statistics Canada, 2017*a*) linked, in 2018, to Ontario Ministry of Health (MOH; formerly MOHLTC) administrative data. Using the 2014 Canadian Child Tax Benefit (CCTB) file as the sampling frame, households were selected based on a complex cluster sample of areas stratified by urban/rural residency and income. The CCTB is a tax system-based family benefit, for which all parents are assessed, creating a roster of all families with children under 18. Data were collected during home visits in 2015 by trained Statistics Canada interviewers. Survey design, content and data collection details are available elsewhere (Boyle *et al*., 2019). Interviewers informed parents that survey data would be combined with administrative data and, with consent, collected a provincial health number to assist with the linkage. Parents were asked for agreement to share their data with the Ontario MOH. A probability sample of 6537 households (50.8% response) participated, with 10 802 children/youth aged 4–17. Of those, 89.5% (*n* = 9666) agreed to share and 96.2% of those were linked to administrative data (*n =* 9301) (sample selection in Fig. 1). Statistics Canada recalculated linked sample survey weights and shared data, weights and health card numbers with the MOH who extracted and provided administrative and survey data to the research team who conducted the linkage.

Ontario administrative health records pertain to inpatient, outpatient and other physician services covered by the Ontario Health Insurance Program (OHIP) which provides nearly universal coverage of Ontario residents (>96%) (Government of Ontario, 2018). Data from 2004 onwards were extracted from the Claims History Database (CHD: physician services billings), Discharge Abstract Database (DAD: inpatient services) and National Ambulatory Care Reporting System (NACRS: day procedures and outpatient services) and combined with the Registered Persons and Client Agency Program Enrolment Databases to determine individual eligibility for OHIP services.

## Measures

### Mental health disorder

The survey uses the DSM-IV-TR (APA, 2000) diagnostic classification system, while administrative data use the 10th revision of the International Statistical Classification of Diseases and Related Health Problems [ICD10; World Health Organization (WHO), 2004] to classify mental disorders, except for CHD data where the 9th revision (ICD9; WHO, 1978) was used. The diagnostic specifications for child/youth mental disorders were largely unchanged between ICD9 and 10. Disorders were selected by aligning groupings across data sources. Given extremely low prevalence in the administrative data, behavioural disorders were dropped. (Only conduct disorder appears in ICD10 and cases were insufficient to meet Statistics Canada disclosure rules.) Final disorder selection includes emotional, consisting of mood and anxiety, attention-deficit/hyperactivity and a group of both emotional and attention-deficit/hyperactivity disorders.

#### Survey data

In families with two or more eligible children, one randomly ‘selected child’ (SC) was assessed on all survey components, including a structured diagnostic interview. The Mini International Neuropsychiatric Interview for Children and Adolescents (MINI-KID) was administered to parents about the SC by trained interviewers to assess disorders in the past 6 months. MINI-KID classifications demonstrate convergent and discriminant construct validity and adequate test-retest reliability across disorders, informants and samples (Sheehan *et al*., 2010; Duncan *et al*., 2018). Disorders included mood (major depressive episode), anxiety (generalised anxiety, separation anxiety, social phobia and specific phobia) and attention-deficit/hyperactivity disorder.

#### Administrative data

Mental disorders were classified according to presence of the corresponding ICD9/10 diagnostic code for an individual in any of the administrative data files in the 6 months preceding the study interview date. Diagnostic codes were identified for mood, depressive, anxiety, emotional and hyperkinetic disorders using case definitions identified by Cairney *et al*. (2015) following code selections (online Supplementary Table A1) that have shown adequate specificity (97%) and sensitivity (81%) in adults (Steele *et al*., 2004). The DAD and NACRS identify fewer disorders than the CHD but were included as eligible sources of disorder identification.

### Mental health-related service contacts

#### Survey data

Service contacts were coded following procedures used by Georgiades *et al*. (2019) to identify physician- and physician office-based services, as this is the only type of service captured in administrative data. Non-physician-based community and school-based services were excluded. Parents were asked whether they had visited different providers and settings for their child/youth's mental health concerns in the past 6 months. Providers included family doctors, paediatricians, other regular health care providers, other types of physicians or specialists, nurses, psychiatrists or other health professionals. Settings included specialised mental health or addictions agencies funded by MOH, walk-in clinics, urgent care facilities and hospital emergency rooms. An indicator of any physician-based mental health-related service contact included endorsement of contact with at least one provider or in at least one service setting.

#### Administrative data

Mental health-related service contacts were identified as any entry in the administrative databases associated with a mental health-related diagnostic classification in the 6 months prior to the child/youth interview date. Diagnostic classifications included disorders beyond emotional and attention disorders as survey-based service contact questions asked about ‘problems children might have with their emotions, attention or behaviour. For adolescents these problems might also include use of alcohol or drugs’. Diagnostic ICD10 classifications included substance-related disorders, schizophrenia, neurodevelopmental and personality disorders, self-harm and eating disorders, and for ICD9 included psychotic, non-psychotic, substance use disorder, social problems and a set of other mental health-related codes that are appropriate when looking at child/youth mental health-related concerns (Cairney *et al*., 2015; Amartey *et al*., 2017).

### Sub-group definitions

We grouped the sample into child (age 4–11) *v*. youth (age 12–17) and male *v*. female based on standard Statistics Canada questions administered to the parent about child/youth age and sex. Calculated based on population size and density, urban–rural residency is a three-category variable of large urban (100 000+), small-medium urban (1000–99 999) and rural residency (<1000 or <400 residents per km^2^) (Statistics Canada, 2017*b*).

### Analysis

To address question 1, we calculated the prevalence in survey and administrative data of individual and grouped mental disorders, and service contacts. One-sample *z*-tests for differences in the estimates were conducted. Simple ratios (not odds ratios) were calculated to quantify the size of the differences between estimates along with bootstrap standard errors and confidence intervals to measure precision. To address question 2, we calculated sensitivity, specificity and agreement between survey and administrative data classifications using Cohen's *κ* coefficient (Cohen, 1960). This is a chance-corrected agreement measure that indicates agreement is slight (*κ* = 0.01–0.20), fair (*κ* = 0.21–0.40), moderate (*κ* = 0.41–0.60) and substantial or excellent (*κ* = 0.61–0.80) (Shrout, 1998). Although sensitivity/specificity usually refer to accuracy in comparison to a gold standard, in our analysis there is none. Sensitivity is the likelihood of a child/youth being classified with disorder/service contact in both the survey and administrative data. Specificity is the likelihood of a child/youth not being classified in neither the survey nor administrative data. To address question 3, we evaluated whether group differences were consistent across data sources, even though overall prevalence might be different. To do this, we calculated prevalence estimates by sub-group and conducted *z*-tests for group differences by age and sex. For geography, joint *χ*^2^ tests were used to identify statistically significant group differences in two-way comparisons. We counted instances where group differences were consistent across data sources. Significant differences were flagged at the *p* < 0.05 level as the least conservative threshold by which group differences are identified.

### Sample for analysis

Analysis was conducted in the representative sample of SCs who were assessed using the MINI-KID (*n* = 5563) linked to administrative data. A complete case analysis was conducted to avoid masking any impact of missing data – a practical consideration when evaluating data source comparability. Sample loss was less than 1% across the linked dataset. Using the sampling weights developed by Statistics Canada adjusted for the likelihood of being linked, population prevalence estimates were calculated. To account for the complex survey design, mean bootstrap weights were applied to produce accurate standard errors.

## Results

Table 1 presents sample characteristics for the shared, linked sample of SCs. There were no differences in characteristics between the full OCHS sample, linked sample and the subsample of SCs (not shown).

**Table 1.** Sample characteristics of linked sample

Sociodemographic characteristics	% (s.e.)
Child *n* = 5563	
Age	
4–11	54.8 (0.67)
12–17	44.2 (0.67)
Sex	
Male	51.5 (0.67)
Female	48.5 (0.67)
Family *n* = 5563	
Single parent family	
Yes	19.6 (0.94)
No	80.4 (0.94)
Household poverty	
Yes	18.6 (0.52)
No	81.4 (0.52)
Urban–rural residency	
Large urban	69.1 (3.02)
Small-medium urban	17.7 (3.27)
Rural	13.2 (1.39)

s.e., standard error.

Table 2 shows estimated prevalence of emotional (mood and anxiety), attention-deficit/hyperactivity disorder and mental health-related service contacts based on survey and administrative data classifications, the results of one-sample *z*-tests and ratios representing how much larger the prevalence estimate is based on survey *v*. administrative data – with values closer to 1 representing prevalence estimates closer in size. Disorder prevalence and contacts are significantly higher in survey data, except for mood disorder, which is significantly but slightly numerically lower (2.87 *v*. 3.57). Ratios are highly variable; the prevalence of emotional disorders is 2.16 times larger based on survey data whereas the prevalence of attention-deficit/hyperactivity disorder is over 11 times larger. For mood disorder, prevalence was 20% lower in survey data, although the 95% confidence interval for the ratio contains 1 indicating this difference may be due to random chance.

**Table 2.** Six-month prevalence of DSM-IV-TR disorders and service contacts based on 2014 OCHS survey data (survey) and administrative health data (admin)

Estimate	Prevalence % (s.e.)		*z* statistic (*p*-value)	Ratio of survey prevalence to administrative data prevalence (s.e.)
	Survey	Admin		
Emotional + attention-deficit/hyperactivity	16.41 (1.20)	5.58 (0.54)	10.35 (<0.001)	2.98 (0.46)
Emotional	11.06 (0.98)	5.10 (0.52)	8.84 (<0.001)	2.16 (0.42)
Mood	2.87 (0.41)	3.57 (0.50)	6.96 (<0.001)	0.80 (0.22)
Anxiety	9.98 (0.96)	2.17 (0.27)	8.52 (<0.001)	4.63 (1.00)
Attention-deficit/hyperactivity	7.58 (0.60)	0.69 (0.12)	10.69 (<0.001)	11.01 (3.40)
Mental health-related service contacts	13.66 (0.84)	7.63 (0.62)	17.56 (<0.001)	1.79 (0.17)

s.e., standard error.

Table 3 presents sensitivity (probability of being classified in both the survey and administrative data), specificity (probability of not being classified in neither survey data nor administrative data) and *κ* coefficients for agreement between data sources. Specificity is high, ranging from 0.98 to 1.00, and sensitivity is low, ranging from 0.07 to 0.41. According to our criteria, agreement ranges from slight (*κ* = 0.13 for attention-deficit/hyperactivity disorder) to moderate (*κ* = 0.46 for service contacts) and average agreement is fair (*κ* = 0.23).

**Table 3.** Concordance between individual disorder classifications and service contacts

Estimate	Sensitivity	Specificity	*κ* (s.e.)
Emotional + attention-deficit/hyperactivity	0.22	0.97	0.27 (0.012)
Emotional	0.19	0.97	0.22 (0.013)
Mood	0.28	0.97	0.16 (0.013)
Anxiety	0.11	0.99	0.14 (0.011)
Attention-deficit/hyperactivity	0.07	1.00	0.13 (0.008)
Mental health-related service contacts	0.41	0.98	0.46 (0.010)

s.e., standard error.

Table 4 presents prevalence estimates by sub-group in survey and administrative data. The larger estimate in statistically significant sub-group comparisons is bolded (age, sex) or flagged (geography). For half of the 18 comparisons, there were no significant group differences in either data source. In the remaining nine comparisons, there were consistent significant group differences in two comparisons – both sex-based (attention-deficit/hyperactivity disorder and service contacts). In these cases, prevalence estimates were double or ten times the size in survey data. There were no consistent age- or geography-based differences in prevalence. Age differences were found for the prevalence of mood and attention-deficit/hyperactivity disorder in survey data, and for anxiety disorder in administrative data. Sex differences were found for mood and emotional + attention-deficit/hyperactivity disorder in administrative data. Differences in prevalence between large urban and small-medium urban geography were identified in survey data for emotional and anxiety disorder.

**Table 4.** Six-month prevalence of DSM-IV-TR disorders by age group and sex based on 2014 OCHS survey data (survey) and administrative health data (admin)

Estimate	Estimated prevalence (%)														
	Age				Sex				Geography						
	Survey		Admin		Survey		Admin		Survey				Admin		
	4–11	12–17	4–11	12–17	Male	Female	Male	Female	LU	SMU	R		LU	SMU	R
Emotional + attention-deficit/hyperactivity	17.13	15.50	4.93	6.39	17.69	15.05	**6**.**72**	4.37	15.09	21.83	15.95		5.20	7.18	5.40
Emotional	9.85	12.62	4.46	5.91	10.08	12.12	6.01	4.13	9.68	16.13	11.35	i	4.68	6.55	5.31
Mood	1.08	**5**.**12**	3.51	3.65	2.91	2.82	**4**.**83**	2.24	2.66	3.65	2.91		3.15	4.96	3.95
Anxiety	9.49	10.60	1.24	**3**.**35**	8.89	11.14	1.95	2.41	5.32	10.45	7.42	i	2.15	1.79	2.79
Attention-deficit/hyperactivity	**9**.**81**	4.75	0.73	0.64	**10**.**81**	4.16	**0**.**96**	0.41	8.48	15.42	10.34		0.74	0.88	0.20
Mental health-related service contacts	13.30	14.12	7.34	8.00	**16**.**18**	10.98	**9**.**02**	6.16	13.30	16.44	11.82		7.55	7.95	7.62

LU, large urban centre; SMU, small-medium urban centre; R, rural.

*Note*: bolded estimates represent the larger estimate in *z*-tests for statistical differences at *p* < 0.05; i = SMU *v*. R *χ*^2^ test significant at *p* < 0.05.

## Discussion

We found differences in disorder prevalence and prevalence patterns across sub-groups, along with slight to fair agreement between individual classifications by data source. Estimates are consistently higher in survey data, suggesting that administrative data underestimate characterisations of mental health need in the population. The magnitude of differences between estimates from the two data sources are inconsistent, making it difficult to draw conclusions about potential systematic bias in survey or administrative data or make recommendations about the possibility for administrative data to replace surveys for certain purposes. More evidence is needed as ours is the first study to date.

Except for mood disorder, overall prevalence estimates were higher in survey data, but the magnitude of the difference varies widely. The higher survey estimates are not surprising given that a selection mechanism for inclusion in administrative data is contact with physician-based services – an important limitation that should be clearly stated and reiterated in child/youth psychiatric epidemiological analysis using only administrative data. Although 70% of children/youth with mental health agency service contacts also report physician-based service contacts (Schraeder *et al*., 2021), some children/youth with disorders are only seen by community or school-based mental health services, and approximately half are not seen by any sector (Georgiades *et al*., 2019). These children/youth are not captured in administrative data which could explain the discordance. However, in a sensitivity analysis where survey data prevalence was calculated, conditional on reports of a physician-based service contact (see online Supplementary Table A2), estimates were slightly lower (emotional + attention-deficit/hyperactivity: by 3.0%, emotional: 2.1%, mood: 0.2%, anxiety: 2.1%, attention: 0.9%) but overall, results were consistent, showing significantly higher estimates in survey data, with the exception of mood disorder. This suggests that the exclusion of disorders counted among children/youth in contact with school or community-based mental health services only partially contributes to observed differences. Other reasons likely include the purpose, process and accuracy of disorder identification, discussed below.

Differences were smaller in disorder groupings and larger for specific disorders, even though common disorders were selected across datasets. This is likely due to different assessment approaches (standardised interview *v*. clinical diagnosis for administrative purposes) and because prevalence estimates will be more similar and likely to agree when disorders are grouped together – for example, mood and anxiety disorders grouped together under emotional disorders.

Prevalence for mood disorder was similar across survey and administrative data (2.9 *v*. 3.6%). However, agreement in individual mood disorder classifications was still low (*κ* = 0.16), which is similar to findings of discordance in adult mood/anxiety disorders between individuals classified by survey *v*. administrative data (Edwards *et al*., 2020; O'Donnell *et al*., 2016). These studies did find differences in prevalence but did not report disorder-specific estimates, so it is unknown if our findings for mood disorder prevalence were consistent. We encourage researchers to report disorder-specific results where possible.

Agreement between disorder classifications was highly variable, with higher agreement observed for grouped disorders. Service contacts demonstrated the highest level of agreement suggesting they are better captured in both sources than mental disorder. The prevalence of disorder and service contacts (<17%) reduces the likelihood of individual agreement (Byrt *et al*., 1993), but agreement was still only fair when disorders were grouped, and selection was harmonised as far as possible. This raises questions about how well these classifications discriminate between disorder types. Findings of low agreement and lack of disorder specificity align with previous evidence examining adult mental disorders (Kisely *et al*., 2009*a*; Edwards *et al*., 2020) and chronic illness (Fortin *et al*., 2017) that point to issues of recording accuracy and completeness of health records in administrative data or that data sources are capturing people at different stages of illness and treatment. Survey data could capture children/youth suffering from clinical levels of symptom severity whose parents have not recognised their need or started seeking physician-based services. Similarly, administrative data could capture children/youth who are being treated and, as a result, may report symptoms at sub-clinical levels in survey data.

Even in the presence of prevalence differences and low agreement, we examined consistency in sub-group differences in prevalence based on child/youth age, sex and geography across data source. Only two sex-based group differences were consistent – both attention-deficit/hyperactivity disorder and service contacts were higher in males than females. There were no consistent age or geography-based differences even though there were sometimes differences in one data source. This suggests that conclusions drawn about key correlates of mental disorder and service contacts will be different and inconsistent within and across groups, depending on data source. This has implications for users of both survey and administrative data who have objectives related to examining between-group differences. Unlike previous studies comparing administrative data to survey self-report (Muggah *et al*., 2013; Payette *et al*., 2020), survey-based disorder classifications are based on a validated, interviewer-administered, standardised diagnostic interview that has demonstrated validity and reliability in general population samples of children/youth. Prevalence estimates produced are consistent with estimates from studies elsewhere (Georgiades *et al*., 2019). Administrative data are subject to reporting bias and inaccuracy in psychiatric diagnoses (Davis *et al*., 2016). Previous adult depression case definition validation efforts have produced suboptimal to moderate levels of sensitivity (Fiest *et al*., 2014; Doktorchik *et al*., 2019). It is unknown whether case definition validation for child/youth mental disorders would produce more accurate estimates – further work is needed. In contrast, we relied on self-reported use of health services, which are notoriously difficult to capture via self-reported questionnaires (Reid *et al*., 2008). Extensive survey-based questions were asked about mental health-related services but the extent to which these questions capture the same contacts as administrative data are likely limited (Rhodes *et al*., 2002). We made every effort to reconcile differences between data sources in disorder selection and focused service contacts on those that were physician-based. Nonetheless, differences remained and were greater for mental disorder than for service contacts.

In the absence of evidence about data source reliability, some researchers are calling for the development of approaches to synthesise disorder classifications across different data sources as a way of improving estimation (Edwards *et al*., 2021; Vigo *et al*., 2021). This could be more appropriate for mental disorders than chronic conditions given unique challenges associated with case definitions. It will be important to determine the strengths of these combined approaches and whether they can be used to examine between-group differences in prevalence in addition to simple prevalence estimation. Linkage of Statistics Canada population surveys like the Canadian Community Health Survey and the 2014 OCHS to administrative data provides opportunities to improve our understanding of the reasons for differences between data sources for certain research objectives and make recommendations about the types of questions that can be answered with these data. Research funder-stipulated data linkage of publicly funded surveys would increase the availability of linked databases, and ongoing linkages are needed to investigate the impact of changes to health services (e.g. changes to virtual services) and diagnosis coding practices on data source difference. Based on our findings, we recommend: (1) focusing on high-level disorder groupings in prevalence estimation; (2) conducting data linkage where possible to access and compare alternate sources of information; and (3) clear reporting of the limited generalisability of administrative data due to disorder inclusion being conditional on contact with physician-based services.

This study is the first to compare prevalence estimates and agreement between child/youth mental disorder classifications and service contacts between general population survey data *v*. linked administrative health data. Study strengths include using a large, linked sample of children/youth within the same health jurisdiction and having access to all available health records. The study also has limitations. First, it is restricted to Ontario, and we know that there is provincial variability in the content and completeness of diagnostic coding (Doyle *et al*., 2020). Replicate work is needed to determine generalisability of our findings. Second, the burden of mental disorder includes symptom severity and complexity, and their impact on child's functioning in daily life and with family/peers (Eaton *et al*., 2008). These factors could determine whether a family seeks health services and surveys may identify individuals whose symptoms are not severely affecting their daily lives. This issue is partially addressed in the current study as the diagnostic interview incorporates questions about the impact of mental health problems into the disorder classification. Nonetheless, more work is needed to determine the mechanisms by which children/youth are identified in each data source. Finally, evidence shows disorder prevalence differences depending on informant (Georgiades *et al*., 2019). This study relies on parent informants in survey data as this allowed for a comparison across an age span of 4–17 years. Agreement between youth-reported survey data and administrative data should also be examined.

## Conclusion

This study compares survey and administrative data sources for estimating child/youth mental disorder prevalence and physician-based mental health-related service contacts – important estimates for policy formulation, research, advocacy and resource allocation. Except for mood disorder, estimates were consistently higher in survey data, and we found variable differences in the prevalence of child/youth mental disorder and service contacts and in age-, sex- and geography-based patterns of prevalence. Individual agreement was, on average, fair and also variable. Our findings highlight the need for users of both survey and administrative data to contextualise their understanding of prevalence of child/youth mental disorder and service contacts based on the data source being used. Further research is needed to understand who and what is being captured by each source. Researchers should conduct data linkage where possible to access and compare multiple sources of information.

## Data Availability

Data access to the 2014 Ontario Child Health Study is available through Statistics Canada Research Data Centres. Data access to the linked dataset will not be shared due to data sharing agreements with Statistics Canada and the Ministry of Health in Ontario.

## Supplementary material

For supplementary material accompanying this paper visit https://doi.org/10.1017/S204579602200018X. click here to view supplementary material
