# Supplementary material for: Estimating prevalence of child and youth mental disorder and mental health-related service contacts: a comparison of survey data and linked administrative health data
Source: Epidemiol Psychiatr Sci. 2022 May 19;31:e35. doi: 10.1017/S204579602200018X (PMC9121846; doi:10.1017/S204579602200018X)
Supplement: Supplementary file 1 [file S204579602200018Xsup001.docx]

Supplementary table A1: Disorder listing

| Disorders | 2014 OCHS (DSM-IV-TR) | NACRS & DAD (ICD10) | OHIP (ICD9) |
| --- | --- | --- | --- |
| Disorders included in mental health related-service contacts variable | N/A | **F10-19, F55** Substance-related disorders  **F20 (excluding F20.4), F22-25, F28, F29, F53.1** Schizophrenia  **F21, F60-62, F68, F69, F80-F84, F88-92, F94, F95, F98** Neurodevelopmental and personality disorders  **F30-F39**: Mood disorders  **F40-48**: Anxiety disorders  **F50.0-50.3, F50.8, F50.9** Eating disorders  **F90** Hyperkinetic disorders  **F92** Mixed disorders of conduct and emotions  **F93** Emotional disorders with onset specific to childhood  **F98.8** Other specified behavioural and emotional disorders with onset usually occurring in childhood and adolescence  **F98.9** Unspecified behavioural and emotional disorders with onset usually occurring in childhood and adolescence  **F99** Mental disorder, not otherwise specified | **291, 292, 299** Alcohol, drug or other psychoses  **295-298** Psychotic disorders  **300** Neurotic disorders (includes reactive depression)  **301, 302, 306, 309** Non-psychotic disorders  **303, 304** Substance use disorders  **307** Habit spasms, tics, anorexia nervosa, sleep disorders  **311** Depressive disorder, not elsewhere classified (Include: depressive disorder NOS, depressive state NOS, depression NOS)  **313** Disturbance of emotions specific to childhood and adolescence  **314** Hyperkinetic syndrome of childhood  **315** Developmental delay  **316** Psychic factors associated with diseases classified elsewhere  **897-902, 904-906, 909** Social problems |
| Emotional+Attention-deficit/hyperactivity | Major depressive episode  Generalized anxiety disorder  Separation anxiety disorder  Social phobia/anxiety disorder  Specific phobia  Attention-deficit/hyperactivity disorder | **F30-F39**: Mood disorders  **F40-48**: Anxiety disorders  **F90** Hyperkinetic disorders  **F92** Mixed disorders of conduct and emotions  **F93** Emotional disorders with onset specific to childhood | **311** Depressive disorder, not elsewhere classified (Include: depressive disorder NOS, depressive state NOS, depression NOS)  **313** Disturbance of emotions specific to childhood and adolescence  **300** Neurotic disorders (includes reactive depression)  **314** Hyperkinetic syndrome of childhood |
| Emotional | Major depressive episode  Generalized anxiety disorder  Separation anxiety disorder  Social phobia/anxiety disorder  Specific phobia | **F30-F39**: Mood disorders  **F40-48**: Anxiety disorders  **F93** Emotional disorders with onset specific to childhood | **311** Depressive disorder, not elsewhere classified (Include: depressive disorder NOS, depressive state NOS, depression NOS)  **313** Disturbance of emotions specific to childhood and adolescence  **300** Neurotic disorders (includes reactive depression) |
| Mood | Major depressive episode | **F30-F39**: Mood disorders | **311** Depressive disorder, not elsewhere classified (Include: depressive disorder NOS, depressive state NOS, depression NOS)  **313** Disturbance of emotions specific to childhood and adolescence |
| Anxiety | Generalized anxiety disorder  Separation anxiety disorder  Social phobia/anxiety disorder  Specific phobia | **F40-48**: Anxiety disorders  **F93** Emotional disorders with onset specific to childhood | **300** Neurotic disorders (includes reactive depression) |
| Attention | Attention-deficit/hyperactivity disorder | **F90** Hyperkinetic disorders | **314** Hyperkinetic syndrome of childhood |

Supplementary table A2: Six-month prevalence of DSM-IV-TR disorders and service contacts based on 2014 OCHS survey data conditional on physician-based service contact (survey) and administrative health data (admin)

| Estimate | Prevalence % (SE) | | *z* statistic  (*p*-value) | Ratio of survey prevalence to administrative data prevalence |
| --- | --- | --- | --- | --- |
|  | Survey | Admin |  |  |
| Emotional+Attention-deficit/hyperactivity | 13.38(1.03) | 5.58 (0.54) | 11.19 (<0.001) | 2.40 |
| Emotional | 8.95 (0.83) | 5.10 (0.52) | 9.78 (<0.001) | 1.75 |
| Mood | 2.67 (0.39) | 3.57 (0.50) | 6.82 (<0.001) | 0.75 |
| Anxiety | 7.91 (0.81) | 2.17 (0.27) | 9.38 (<0.001) | 3.64 |
| Attention-deficit/hyperactivity | 6.64 (0.0.56) | 0.69 (0.12) | 10.66 (<0.001) | 9.62 |

SE=standard error
